# Word learning reveals white matter plasticity in preschool children

**DOI:** 10.1007/s00429-020-02024-7

**Published:** 2020-02-18

**Authors:** Clara E. M. Ekerdt, Clara Kühn, Alfred Anwander, Jens Brauer, Angela D. Friederici

**Affiliations:** Department of Neuropsychology, Max Planck Institute for Human Cognitive and Brain Sciences, Stephanstr. 1a, 04103 Leipzig, Germany

**Keywords:** White matter, Plasticity, Development, Language acquisition, Diffusion tensor imaging (DTI)

## Abstract

Word learning plays a central role in language development and is a key predictor for later academic success. The underlying neural basis of successful word learning in children is still unknown. Here, we took advantage of the opportunity afforded by diffusion-weighted magnetic resonance imaging to investigate neural plasticity in the white matter of typically developing preschool children as they learn words. We demonstrate that after 3 weeks of word learning, children showed significantly larger increases of fractional anisotropy (FA) in the left precentral white matter compared to two control groups. Average training accuracy was correlated with FA change in the white matter underlying the left dorsal postcentral gyrus, with children who learned more slowly showing larger FA increases in this region. Moreover, we found that the status of white matter in the left middle temporal gyrus, assumed to support semantic processes, is predictive for early stages of word learning. Our findings provide the first evidence for white matter plasticity following word learning in preschool children. The present results on learning novel words in children point to a key involvement of the left fronto-parietal fiber connection, known to be implicated in top-down attention as well as working memory. While working memory and attention have been discussed to participate in word learning in children, our training study provides evidence that the neural structure supporting these cognitive processes plays a direct role in word learning.

**Electronic supplementary material:**

The online version of this article (10.1007/s00429-020-02024-7) contains supplementary material, which is available to authorized users.

## Introduction

Learning single words is the way children break into the human communication system, and the number of words a child knows is a key predictor for later academic success (Morgan et al. 2015; Bleses et al. 2016). With the significant developmental relevance of word learning, the neural underpinnings of this process are of great interest. The goal of the present study is to investigate the white matter structures involved in word learning in preschool-aged children by measuring possible structural changes in the brain as a consequence of word learning.

In adults, word learning initially occurs as an episodic event supported by the hippocampus (Davis and Gaskell 2009). After consolidation, the new lexical entry becomes part of the lexicon represented throughout the neocortex. Recent results coming from studies using several different methods including magnetoencephalography (MEG), as well as functional and structural magnetic resonance imaging (MRI), have shed light on this process in adults (Hofstetter et al. 2017; Bakker-Marshall et al. 2018; Berens et al. 2018). One study has shown that in adults, word learning leads to white matter changes in regions of the language network. After 1 h of word learning, structural changes in the white matter of the parietal lobe were found and changes in the superior longitudinal fascicle correlated with the lexical learning rate (Hofstetter et al. 2017).

Training studies provide a method to study the brain structures that are involved in the functional process under investigation (Schlegel et al. 2012). A seminal study in the motor domain, measuring brain structure changes before and after adults learned to juggle, observed gray matter changes in regions supporting the perception of objects in motion and the anticipation of where they would be next (Draganski et al. 2004). Focusing on white matter, Scholz et al. (2009) found changes in white matter microstructure after 6 weeks of juggling training. Quickly thereafter, additional evidence for white matter plasticity in adults, measured in vivo using MRI, was found following different types of interventions ranging from motor-based to cognitive functions, such as word learning (Mackey et al. 2012; Taubert et al. 2012; Salminen et al. 2016; Hofstetter et al. 2017).

Functionally, children are already expert word learners by the age of 4 years. Starting around the age of 18–24 months, the speed of word acquisition increases considerably and many studies have shown that children need very few pairings between a new object and its new label to be able to make the link between the two (Carey and Bartlett 1978; Bloom and Markson 1998; Horst and Samuelson 2008; Friedrich and Friederici 2011). The process of word learning is thought to require language domain-specific processes such as semantics (Friedrich and Friederici 2008), and moreover, domain-general processes such as working memory (Baddeley et al. 1998) and attention (Majerus et al. 2009). It has been shown that children with poor phonological memory ability perform worse on a novel word learning task than their peers with high phonological memory scores both in the immediate learning tests as well as on recall tests 1 day later (Gathercole and Baddeley 1990). While the mechanisms important for word learning in preschool children have been well described behaviorally, the underlying neural structures implicated in word learning remain to be elucidated. Here, we implemented a training study to probe the white matter structures involved in word learning in young children in order to shed further light on this open question.

While white matter plasticity can be detected and localized using diffusion MRI (dMRI), the precise physiological mechanisms that underlie structural changes in white matter following training cannot be determined using dMRI. Candidate processes of the different ways in which myelin surrounding the axons can adapt to meet the new processing demands placed on the brain to succeed in the trained task at hand are outlined in a recent review (Kaller et al. 2017). These are myelination of a previously unmyelinated axon, replacement of existing myelin, increase or decrease in the size of the myelin sheath, both in width and in length, as well as change in the spacing between the nodes of Ranvier (Kaller et al. 2017). Fractional anisotropy (FA), a diffusion MRI-based measure of directionality of water diffusion, can be used to assess microstructural changes of white matter (Lebel and Beaulieu 2011), including myelination, and is a measure commonly used in training studies investigating white matter changes (e.g., Scholz et al. 2009; Salminen et al. 2016). Due to the well-demonstrated sensitivity of FA to measure structural changes in the white matter, here, we assessed FA change to elucidate the white matter structures involved in word learning in children.

Currently, it is debated whether the behavioral outcome of a training intervention can be related to the measured white matter structural changes. While some studies report a correlation between structural changes in white matter and training performance (e.g., Hofstetter et al. 2017), others find no such correlation between the structural change and a training outcome measure (Scholz et al. 2009; Chavan et al. 2015), arguing that the structural changes observed reflect time spent on the trained task rather than what was achieved during this training. Interestingly, while there are several studies that find either a relationship in which performance correlates positively with the structural changes in the white matter or no such relationship, at least one study reported a negative correlation between structural changes and training performance in young adults (Taubert et al. 2010). The variation in the results makes it evident that while the training literature has grown rapidly in recent years, a number of questions concerning the physiological mechanisms underlying white matter changes remain unanswered. Here, we chose an exploratory approach to investigate the potential relationship between white matter changes and behavioral changes in children.

In the current study, we took advantage of the possibility to measure white matter changes in vivo under controlled conditions in children. We analyzed changes in fiber connections induced by training as a function of word learning in the developing brain. On the one hand, changes could be expected in regions of the language network, possibly similar to those found in adults (Hofstetter et al. 2017) or in semantic-related regions in the temporal lobe. On the other hand, since children were previously found to recruit additional brain regions and connections to complete language tasks efficiently (Walton et al. 2018), one might also expect changes in regions that play a supporting role in successful word learning such as working memory and attention, as these are indispensable to the process of word learning.

A small, but growing body of literature has probed the possibility to employ training-related white matter plasticity in children. Most of these studies have focused on school-aged children with difficulties in instruction-based skills such as reading or spelling (Keller et al. 2009; Gebauer et al. 2012; Jolles et al. 2015; Huber et al. 2018). After intensive remedial training in children with poor reading skills, FA changes were detected in the left anterior centrum semiovale, and this FA change was correlated with phonological decoding, a process central to reading (Keller and Just 2009). In contrast to this focal change in white matter structure, children between the ages of 7 and 12 years participating in 8 weeks of intensive, daily reading-related training displayed changes in the microstructural properties of white matter in the ventrally located inferior longitudinal fascicle (ILF) and the dorsally located arcuate fascicle (AF), as well as changes in other white matter structures that correlate with the ILF and AF changes, respectively (Huber et al. 2018). These previous studies have tested plasticity in school-age children in order to find evidence of training and the related brain structural changes. Only one of these studies, however, investigated plasticity following training in typically developing children (Jolles et al. 2015). Consequently, in the training literature up to now, two important issues are still left unaddressed. First, to our knowledge, there is no investigation on how word learning changes the brain’s white matter structure following training in preschool children. Second, it is unclear to what extent changes in the white matter can be related to word learning ability and whether white matter microstructure is predictive of word learning success.

Therefore, we designed a study to investigate whether a short period of increased word learning in preschool children would lead to FA changes in their brains’ white matter. To rule out alternate reasons for potential FA changes, we included a passive control group to account for FA changes related to maturation, and an active control group to test the specificity of potential FA changes. Based on previous findings of word learning leading to white matter plasticity in adults (Hofstetter et al. 2017), we expected FA changes in the word learning group. As a next step, we tested whether changes in FA were related to word learning ability. Finally, we assessed to what extent word learning ability could be predicted by white matter microstructure before learning. White matter microstructure has previously been linked to individual differences in language-related developing abilities in children (Walton et al. 2018), and therefore, we expected white matter microstructure to be predictive of word learning in the children in our sample.

Here, we show that after 3 weeks of learning novel words for novel objects, typically developing preschool-aged children demonstrate changes in white matter microstructure, that the change in FA is related to word learning success, and finally, that white matter structure plays a predictive role in explaining individual differences in word learning ability in young children.

## Materials and methods

We conducted a language training study with 4-year-old children who were pseudo-randomly assigned either to a word learning group, an active control group, or a passive control group. We acquired diffusion-weighted magnetic resonance images before and after a 3-week period of language training, see Supplementary Fig. 1 for an overview of the sessions completed by participants. The training group learned 60 new pseudo-words in a picture-matching task during the training period. The active control group completed a sentence–picture-matching task using the same experimental setup as the training group. The passive control group received no intervention and only participated in the MRI acquisition sessions. These data were collected as part of a larger longitudinal project in which the children completed a third MRI scan, 3 weeks after the second MRI scan. We did not have enough usable data to include the third scan in this analysis.

### Participants

Fifty-nine 4-year-old children were included in the analyses: 20 in the word learning group (12 male, mean age = 54.60 months, SD = 4.23 months), 19 in the active control group (10 male, mean age = 51.53, SD = 4.53), and 20 in the passive control group (9 male, mean age = 52.00, SD = 3.91); statistical comparisons of groups for age and gender are reported in Table 1. Children who participated in this study were typically developing, monolingual German-speaking children from a medium-sized German city. 111 children met the inclusion criteria of no previous neurological complications, passing a mock scan session, and completing at least six behavioral sessions in the case of the word learning and active control groups. A total of 52 children were excluded from further data analysis for the following reasons: left handedness (*n* = 3), insufficient data quality due to movement in the scanner or scanner artifact (*n* = 28), missing the second scan (*n* = 11), incidental findings during study MRI scan (*n* = 6), or not reaching above-chance performance in both the last training and posttest in the word learning group (*n* = 4). The procedure of the experiment was explained to the participant’s parents or guardians, who then gave written informed consent. The children gave verbal assent to participate in the study. Parents or guardians received a small remuneration (7.50€) per visit to the research institute. Children received small gifts at the end of each visit to the research institute and received stickers after each training session at their preschool. The study was approved by the ethics committee of the Medical Faculty of the University of Leipzig in accordance with the Declaration of Helsinki.

**Table 1:** Participant characteristics, split by group

	Word learning	Active control	Passive control	Group statistics	
Gender (female/male)	8/12	9/10	11/9	*χ*^2^(2, *n* = 59) = 0.90	*p* = 0.67
Age at scan 1 (months)	54.60 (4.24)	51.53 (4.53)	52.00 (3.91)	*H*(2) = 5.56	*p* = 0.06
TSVK T-score	56.55 (18.74)	69.00 (16.95)	61.95 (17.66)	*F*(2,56) = 2.34	*p* = 0.10
AWST-R T-score	48.90 (7.61)	54.37 (7.96)	50.50 (6.00)	*F*(2,56) = 2.93	*p* = 0.06
Digit Span Scaled Score	9.25 (2.49)	9.89 (2.42)	8.90 (1.80)	*H*(2) = 1.42	*p* = 0.50
Mottier (number of nonwords)	13.26 (6.06)	14.50 (5.72)	13.17 (5.24)	*F*(2,52) = 0.31	*p* = 0.74
Handedness LQ	55.47 (24.46)	48.53 (30.03)	45.94 (33.58)	*F*(2,51) = 0.52	*p* = 0.60
WPPSI Full-scale IQ	97.45 (10.44)	99.11 (8.67)	98.60 (12.38)	*F*(2,56) = 0.12	*p* = 0.88
Days between scans	27.75 (2.43)	28.89 (2.00)	28.60 (2.91)	*H*(2) = 2.82	*p* = 0.24
Mean FA change (on skeleton)	0.002 (0.003)	0.001 (0.004)	0.002 (0.003)	*F*(2,56) = 0.31	*p* = 0.74

Values for all variables aside from gender are mean and (standard deviation). Chi-squared test is reported for gender. Kruskal–Wallis rank sum test results are reported for age, digit span, and days between scans since the assumptions for an ANOVA were not met for these variables. For all other variables, results from ANOVA are reported

### Stimuli

**Fig. 1:**
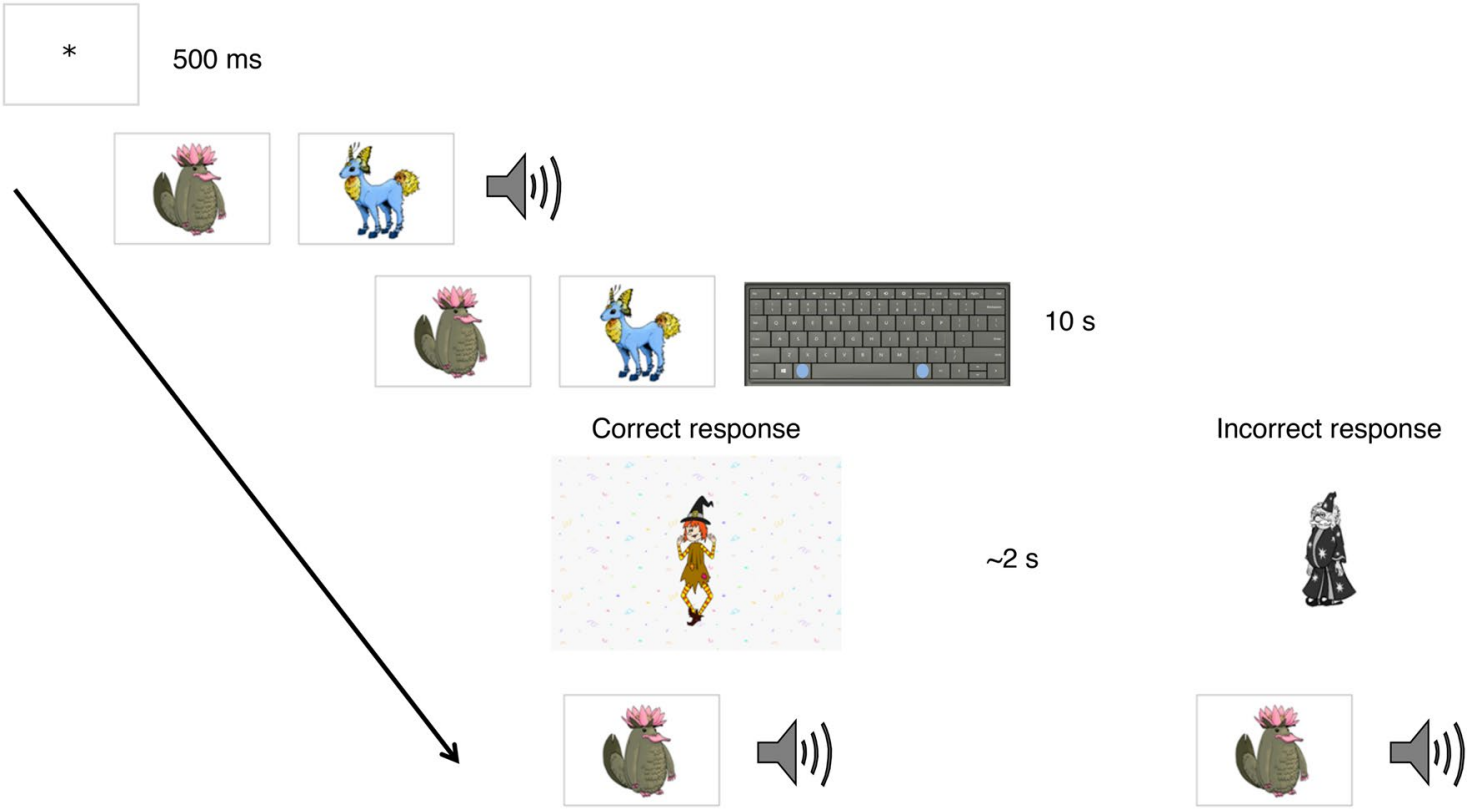
Example of word learning training trial. During each of eight training sessions, children completed 60 trials during which all pseudo-animal—label pairs were presented. The trial started with a fixation trial for 500 ms. Next, two pseudo-animals were presented visually side by side and one pseudo-word was presented auditorily. After the auditory presentation of the pseudo-word, the two images remained on the screen and children had up to 10 s to choose the animal that corresponded to the pseudo-word they had just heard. To choose the animal on the left of the screen, the children were instructed to press the button to the left of the space bar, and for the animal on the right, the button to the right of the space bar. The two response keys were marked with stickers. If the child chose the pseudo-animal that matched the pseudo-word they heard, they saw a video in which a cartoon character did a small celebratory dance. In case of the wrong selection, the children saw a black and white cartoon character that was sad. These feedback videos lasted approximately 2 s. Regardless of whether the participants’ response was correct or incorrect, after the feedback video, only the correct pseudo-animal was presented visually in the middle of the screen, and the corresponding pseudo-word was presented auditorily again. After this, the next trial began. The order of items was different in each session

The novel words to be learned by the word learning group were labels of 60 pseudo-animals, see Fig. 1 for an example of a pseudo-animal. The labels were three-syllable pseudo-words made up of German phonology-permissible syllables (e.g., Speseibe). For each of the 60 pseudo-animals, the children saw six different images, depicting pseudo-animals at different angles or performing different actions (e.g., flying, running). The labels were recorded by a native German-speaking female speaker in a child-directed voice and presented auditorily during the experiment.

### Procedure

The study was conducted over 6 weeks (ref. Supplementary Fig. 1). The children in the word learning group and the active control group participated in one mock scan session, two MRI sessions, eight word learning or sentence comprehension sessions respectively, and one IQ testing session. The children in the passive control group participated in one mock scan session, two MRI sessions, and one IQ testing session. During the first visit, children were acquainted with the MR environment using a mock scanner and completed a standardized test battery assessing language abilities, such as vocabulary (Aktiver Wortschatztest für 3-bis 5-jährige Kinder (AWST-R), Kiese-Himmel 2005), and sentence comprehension (Test zum Satzverstehen von Kindern (TSVK), Siegmüller et al. 2010), as well as working memory (Digit Span Forward, Kaufman et al. 2003; Mottier, Kiese-Himmel and Risse 2009), and handedness (adapted Edinburgh inventory, Oldfield 1971). Due to scheduling constraints, IQ (Wechsler preschool and primary scale of intelligence-Third edition (WPPSI-III), Petermann 2009) was tested 6 months after the first MRI session.

After a successful mock scan session, children participated in the first MRI scan. During all MRI scans, children watched a movie of their choice. After the MRI, all children were read a story to frame the upcoming tasks in a child-friendly way. Children in the word learning group completed a familiarization session with the training material, and children in the active and passive control groups completed a sentence comprehension task. Tasks were completed on a laptop using the software Presentation (Neurobehavioral Systems, Inc., https://www.neurobs.com/) for stimulus delivery and external speakers. Response keys were to the left and the right of the space bar on the keyboard. During the familiarization session, the word learning group was introduced to the pseudo-animals and the matching pseudo-word label. Children saw the pseudo-animal’s picture and heard the correct spoken label repeated twice each, no response was required. Each familiarization trial lasted 4 s.

Over the following 3 weeks, children in the word learning group participated in eight sessions of word learning. These sessions were administered to each child individually and took place at the child's preschool in a quiet room. During each trial, the child saw two pseudo-animals, heard one label, and had to choose the pseudo-animal that matched the label. Response time was restricted to 10 s. Upon response, the child received corrective visual feedback and was then presented with the correct pairing of pseudo-animal and label (see Fig. 1 for an example trial). Each training session consisted of 60 trials, during which each pseudo-animal appeared once as a target and once as a foil, and lasted approximately 10–15 min, including breaks. The representation and order of items presented was different in each training session.

Children in the active control group performed eight sessions of a sentence comprehension task. While they were presented with pictures of two agent–patient relationship scenes, they heard a subject or object relative sentence and had to choose the picture depicting the action described in the sentence. In one picture, the agent–patient relationship matched the heard sentence; in the other, the agent–patient roles were reversed. The children received corrective visual feedback to their response. The agents and patients in the pictures were cartoon renderings of animals well known to 4-year-old children (e.g., dog, bear, tiger). The passive control group received no experimental intervention during these 3 weeks.

During the week after the intervention period, a second MRI scan was administered. Directly after the MRI scan, the children in the word learning group completed a posttest. The posttest session in the word learning group was identical to the word learning trials during training (Fig. 1), except that there was no feedback. The two control groups completed the same sentence comprehension task as after the first MRI scan. This is the same task as the sentence comprehension task completed by the active control group during the intervention period, save for two changes. The sentence comprehension task that the active and passive control groups completed after the first and second scan did not have feedback, and in addition to subject and object relative sentences, this task included simple subject and object first sentences.

### Neuroimaging data acquisition

MR images were acquired on a Siemens Tim Trio 3 T Scanner (Siemens Healthcare, Erlangen, Germany) using a 32-channel head coil, while children watched cartoons on MR-compatible video goggles and headphones. During the two scanning sessions, we collected an anatomical MP2RAGE image (1.2 × 1.0 × 1.0 mm resolution; TR = 5 s; TE = 3.24 ms; GRAPPA 3; acquisition time = 5 min 22 s) (Marques et al. 2010) and whole-brain diffusion-weighted images. We acquired 60 diffusion-weighted images (*b* = 1000 s/mm^2^) and 7 non-diffusion-weighted images (*b* = 0 s/mm^2^) using a multiplexed echo planar imaging sequence (1.9 mm isotropic resolution; TR = 4 s; TE = 76 ms; 66 slices; GRAPPA 2; simultaneous multi-slice 2; partial Fourier 7/8; monopolar diffusion gradient; acquisition time = 5 min 23 s) (Feinberg et al. 2010; Moeller et al. 2010). During the first scanning session, we also collected a FLAIR image to exclude incidental findings.

### Data analysis

Possible differences between groups in demographic variables were tested with Kruskal–Wallis rank sum tests, where necessary, one-way ANOVAs and Chi-squared tests in R version 3.3.1 (R Core Team 2013), see Table 1 for descriptive statistics and statistical tests.

Before preprocessing, we inspected the raw diffusion data for imaging artifacts and excluded volumes from the analysis that were corrupted by motion. The number of volumes removed did not differ between groups or across the two scan timepoints. Only children for whom no more than ten volumes were removed were included in the analyses. All children in the analyses had adequate image quality for both scans. Images were preprocessed using ExploreDTI (Leemans et al. 2009). Signal drift correction followed by subject motion, eddy current and magnetic susceptibility correction were carried out, and FA maps were calculated using the REKINDLE algorithm (Leemans and Jones 2009; Irfanoglu et al. 2012; Tax et al. 2015).

Using Advanced Normalization Tools (ANTs, Avants et al. 2008), we created within-subject templates from FA maps of scan 1 and scan 2 for all included participants. The individual templates were then used as input for creating a group-specific template with ANTs. Next, we registered the FA maps from scan 1 and scan 2 to the individual templates and created the mean of these two registered FA maps. This procedure is similar to that employed in a previous training study (Scholz et al. 2009).

We compared the differences between groups in FA change using tract-based spatial statistics (TBSS, Smith et al. 2006). The mean of the registered scan 1 and scan 2 FA maps was used as input for the initial registration onto the group-specific template, skeletonization and projection in the TBSS analysis using the default TBSS pipeline. This included the computation of the projection of the FA values from each individual’s mean FA to the group template. To use the same projection for the two time points of each participant, we then projected FA values onto the skeleton from the FA maps from scan 1 and scan 2 that had been registered to the within-subject template.

We examined the alignment of FA maps from scan 1 and scan 2 within participants to rule out that any potential group differences in FA change were driven by misalignment. To do so, we calculated the correlation between the FA maps from scan 1 and scan 2 for each participant using AFNI’s (Cox 1996) 3ddot. After performing a Fisher-*z* transformation of these correlation coefficients, we then tested for group differences in alignment using an ANOVA. Both the Fisher-*z* transformation and the ANOVA were conducted in R.

### Statistical analysis

The difference map of each participant was created by subtracting the skeletonized FA map of scan 1 from the skeletonized FA map of scan 2 (post-training–pre-training). Using non-parametric permutation testing (randomise) (Winkler et al. 2014), we tested for differences between the groups on these difference maps with an *F*-test. We included the number of days between the two scans, the age at scan 1, and gender as covariates of no interest in the analysis. To assess which group was driving the potential group differences as indicated by the *F*-test, we conducted post hoc *t*-tests comparing the word learning group to the active and passive control groups separately, as well as comparing the active and the passive control groups directly. The *t*-tests were also conducted using randomise to implement non-parametric permutation testing. This was done within the mask of voxels that showed a significant group effect as previously revealed by the *F*-test.

Correlations between FA and behavioral outcomes of the word learning training were also calculated using randomise on the skeletonized FA data. We conducted separate correlation analyses for two FA measures (FA change and Scan 1 FA), with two behavioral measures [(a) accuracy at training session 1 and (b) average accuracy of all training sessions]. The accuracy of training session 1 (a) reflects the amount of information the children were able to remember after just one session of familiarization with the new items. The average accuracy over all trainings measure (b) captures the speed with which children learn the link between object and label, since children who already do well during early training sessions will have a higher average accuracy than children performing poorly in the first training sessions. We preferred this measure over the difference between performance at training 1 and performance at training 8, since the difference penalizes children who performed very well at the first training, as they do not have a chance to show a large gain in performance due to the ceiling performance.

All results were corrected for multiple comparisons. The group comparison analysis was corrected with threshold-free cluster enhancement (TFCE) (Smith and Nichols 2009), and the brain-behavior correlations were corrected with a cluster-size correction using AFNI's (version 17.0.04) 3dFWHMx and 3dClustSim which has previously been used to relate behavior to FA and other tensor-derived indices (Grosse Wiesmann et al. 2017; Neef et al. 2018). The cluster-forming threshold was *p* < 0.01, and cluster size was significantly larger than needed for the *p* < 0.05 level.

## Results

The three participant groups did not differ significantly in age, full-scale IQ, measures of vocabulary, sentence comprehension or working memory (*p*s > 0.05). The days between MRI scan 1 and scan 2 were not different between the three groups (*p* = 0.33), means, standard deviations, and results of group comparisons are listed in Table 1. The three groups did not differ in parental education [*F*(2,56) = 2.23, *p* = 0.12] which we used as a proxy for socio-economic status. We calculated an index of parental education by averaging both the type of high school diploma that the parents had received as well as any further qualification they received in tertiary education (Kuhl et al. 2020).

### Behavior

**Fig. 2:**
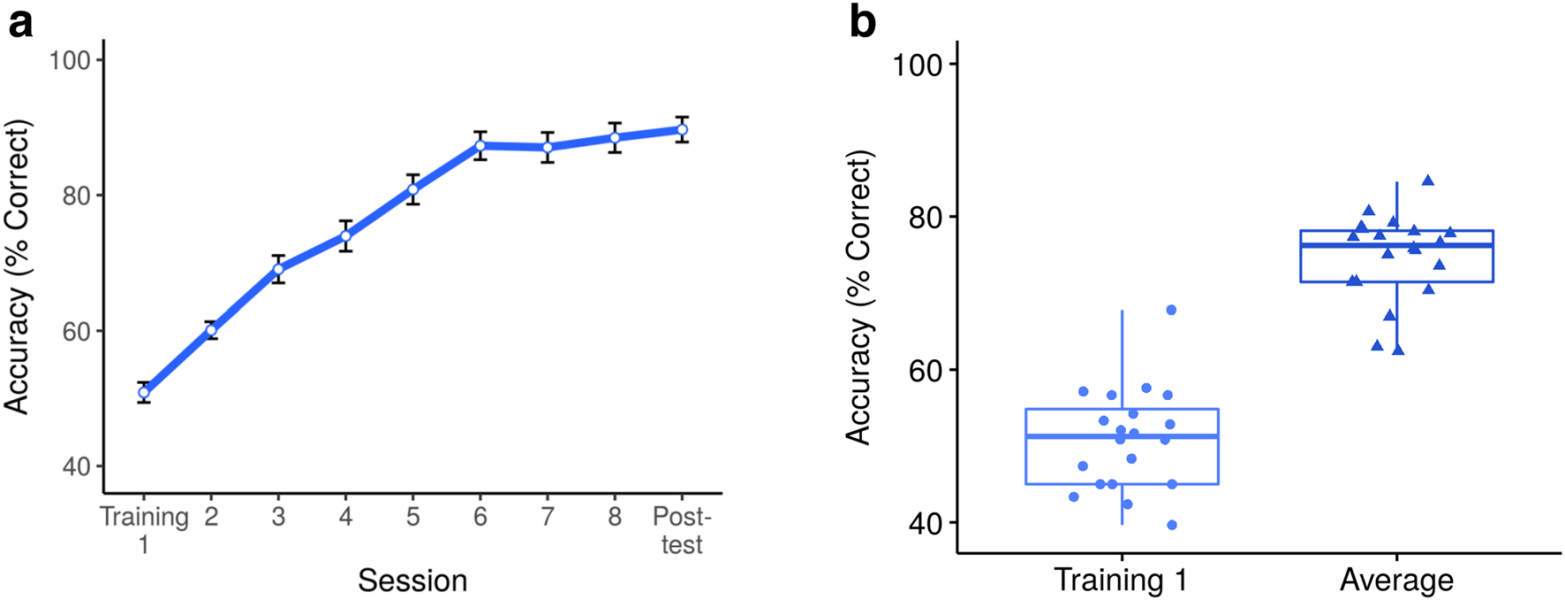
Behavioral results of word learning training **a** Word learning accuracies during training sessions 1–8 as well as posttest averaged across the word learning group. Error bars ± 1 SE. **b** Box plots showing medians for variables used in brain–behavior correlations. Individual subjects' data points are represented by dots in training session 1 and by triangles in the average training accuracy

The children excelled at the word learning task (Fig. 2). The word learning group reached above-chance performance (50%) in accuracy of correctly matching the unknown pseudo-animals and their novel labels in the second training session, *p* < 0.001. In all following sessions, the group performed significantly above chance. The group average accuracy did not significantly increase after the sixth training session (Table 2).

**Table 2:** Behavioral outcome of word leaning group

	Mean	SD	Min	Max
Training 1	0.51	0.07	0.40	0.68
Training 2	0.60	0.06	0.50	0.73
Training 3	0.69	0.09	0.48	0.87
Training 4	0.74	0.10	0.54	0.98
Training 5	0.81	0.10	0.56	0.97
Training 6	0.87	0.09	0.61	0.98
Training 7	0.87	0.10	0.63	0.98
Training 8	0.88	0.10	0.67	0.98
Posttest	0.90	0.08	0.68	0.98

Descriptive statistics for behavioral outcome of word learning group, proportion correct, button presses only. *SD* standard deviation

### Structural neuroimaging

Alignment between scan 1 and scan 2 FA maps did not differ significantly between groups, *F*(2,56) = 0.76, *p* = 0.47.

**Fig. 3:**
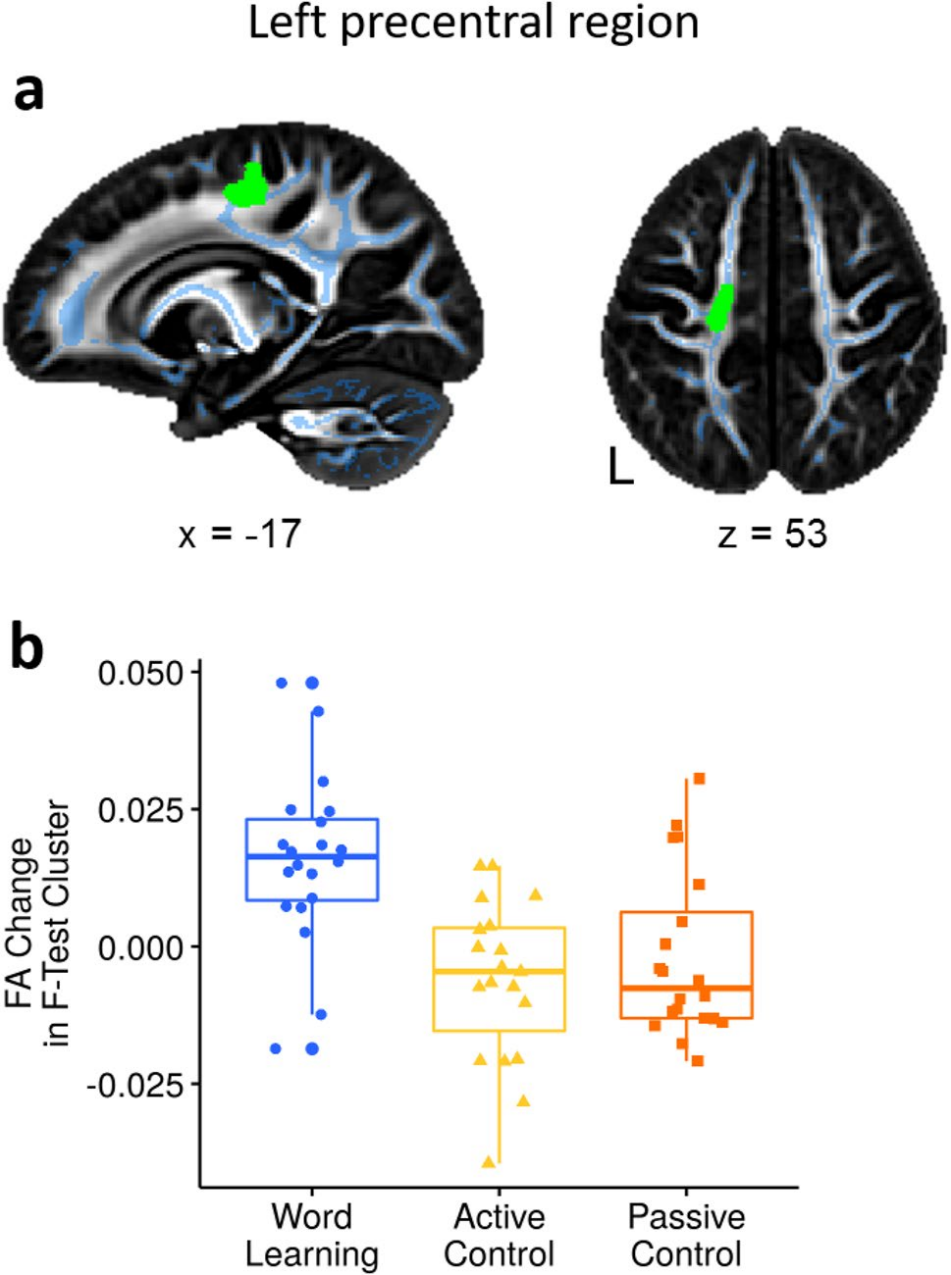
Brain group difference results **a** FA change on the skeleton tested for differences from scan 1 to scan 2 between groups. Significant cluster (green) in the white matter below the precentral gyrus shows greater FA change from scan 1 to scan 2 in the word learning group compared to active and passive control groups. For visualization purposes, the significant clusters were thickened to fill in the local white matter. **b** Plot depicts mean FA change from scan 1 to scan 2 in each group extracted from the significant cluster. Individual subjects' data points are represented by dots in the word learning group, triangles in the active, and squares in the passive control group

We tested for a difference in FA change from scan 1 to scan 2 between the word learning, the active, and the passive control groups using an *F*-test. This analysis was controlled for age, gender, and days between scans. We report differences in the change in FA from scan 1 to scan 2 between the three groups in the left hemisphere in the white matter underlying the dorsal precentral gyrus, see Fig. 3a. Two clusters in this region were significant (MNI: − 17, − 19, 53, k = 53; *F* = 6.84, *p* < 0.05, TFCE corrected and MNI: − 16, − 10, 55, k = 16; *F* = 6.86, *p* < 0.05, TFCE corrected). Extracted FA values from the significant clusters are shown in Fig. 3b for all three groups. This area is a zone of crossing fibers from the corpus callosum, cortico-spinal tract, and the most dorsal part of the superior longitudinal fascicle (SLF I). The three groups did not differ in global mean FA change on the whole skeleton from scan 1 to scan 2, statistical test results are reported in Table 1 (see also Supplementary Fig. 2, Online Resource 1).

We then conducted post hoc *t*-tests to determine which groups differ in FA change in this region. We conducted six *t*-tests, using the *F*-test result as a mask for the analysis. This analysis revealed that the word learning group showed a larger FA change in this region than both the active and the passive control groups (TFCE corrected across voxels, and Bonferroni corrected across tests).

### Correlation of white matter microstructure with behavior

**Fig. 4:**
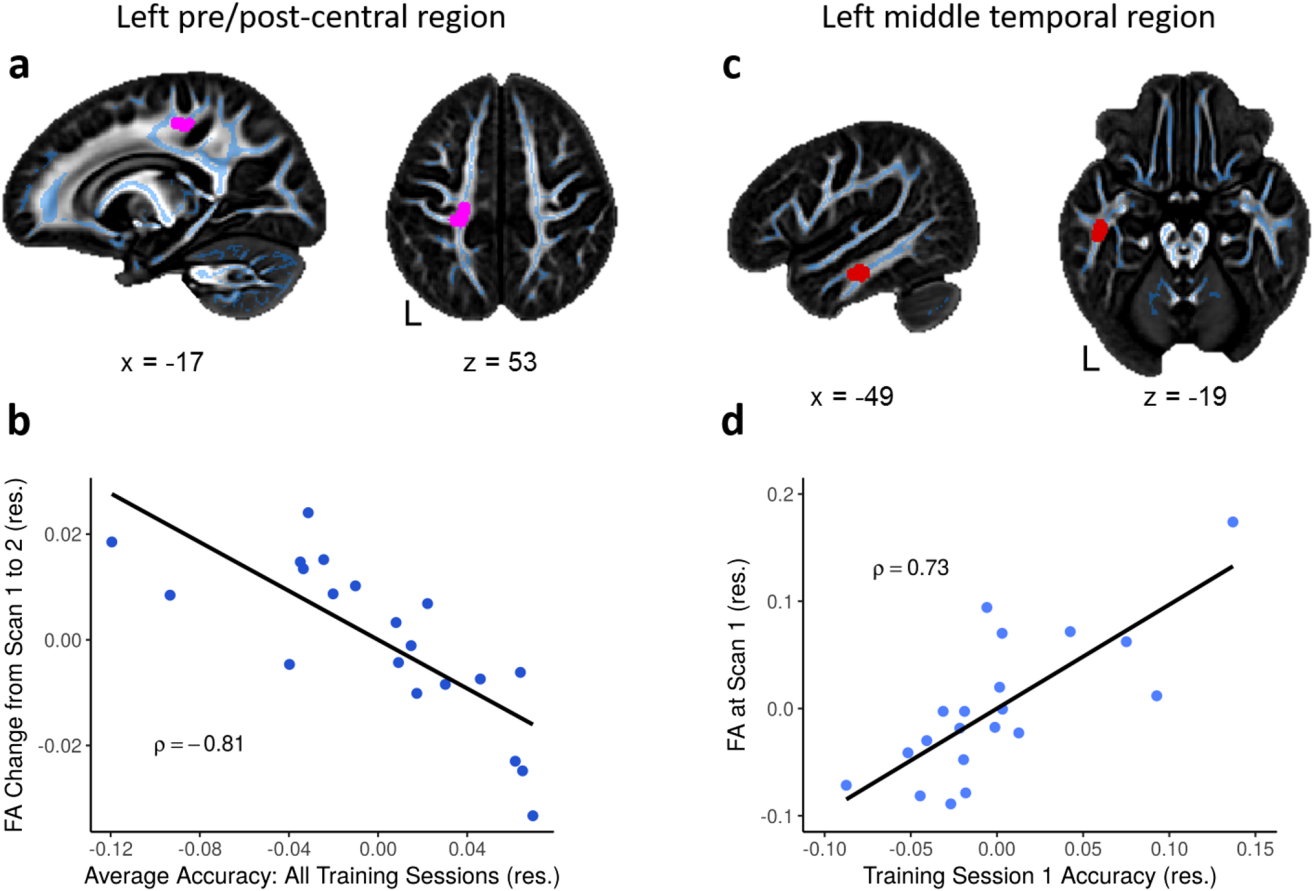
Brain-behavior correlation **a** significant negative correlation between FA change from scan 1 to scan 2 and total average word learning accuracy in left postcentral white matter (in pink). For visualization purposes, the significant clusters were thickened to fill in the local white matter. **b** Extracted FA difference values from significant cluster in FA change × average accuracy correlation plotted against average accuracy scores. **c** Significant positive correlation between FA at scan 1 and word learning training session 1 accuracy in the left middle temporal white matter (in red). **d** Extracted scan 1 FA values from significant cluster in Scan 1 FA × Training 1 accuracy correlation plotted against the accuracy from training session 1. In **b** and **d**, Spearman’s rho is indicated (*ρ*), and values plotted are standardized residuals

Next, we analyzed whether FA in the word learning group was related to the behavioral outcome of the word learning intervention. We conducted correlation analyses to determine whether there was a relationship between FA change from scan 1 to scan 2 with (a) accuracy at training session 1 or (b) the average accuracy of all training sessions. There was no significant correlation between FA change from scan 1 to scan 2 and the accuracy at training session 1. The correlation between FA change and average training accuracy showed a significant negative correlation in the left postcentral white matter, Fig. 4a, (MNI: − 23, − 31, 46; k = 41; *t* = 3.37, *p* < 0.01, cluster-size corrected). Figure 4b displays the extracted FA difference values from the significant cluster plotted against total average accuracy. The correlation between FA change and the average accuracy of all training sessions indicates that children who take longer to learn the new words (lower mean performance) show a greater FA increase in this region.

Additionally, we were interested in whether FA at scan 1 predicted word learning success. We correlated FA at scan 1 (a) with the accuracy at training session 1 and (b) with the average accuracy of all trainings. These analyses showed a positive correlation between FA at scan 1 and accuracy in training session 1 in the white matter located in left middle temporal cortex, Fig. 4c (MNI: − 49, − 20, − 19; k = 38; *t* = 3.30, *p* < 0.01, cluster-size corrected). Extracted scan 1 FA values from the significant cluster are plotted against the accuracy of training session 1 in Fig. 4d. The correlation between FA at scan 1 and the behavioral outcome of training session 1 indicates that children who have higher FA values in the left temporal white matter at the outset will remember more words after just one exposure to a pseudo-animal–word pair. The correlation between FA at scan 1 and average accuracy across all trainings was not significant.

## Discussion

In the current study, we investigated word learning in 4-year-old children and the related changes in the microstructure of the cerebral white matter. While previous studies in adults reported white matter structural changes after word learning (Hofstetter et al. 2017), to date, this has not been demonstrated in children. Here, we report first evidence of white matter structural change following training in preschool-aged children in the domain of word learning. Based on previous findings of training studies in adults (Hofstetter et al. 2017), we expected changes in white matter microstructure as a function of training in the word learning group in young children. When comparing the change in FA between the word learning group, and the active and passive control groups, we found that the word learning group showed a significantly larger FA increase in the left precentral white matter than the two control groups. This change cannot be explained by maturation alone, as the reported change is tested against a passive control group. Importantly, this change can also not solely be explained by the additional interaction the children had with experimenters, nor the interaction with the experimental setup, since the reported change is different also from an active control group for whom the experimental setup but not the learning content was the same. The present findings are the first to show word learning-related brain plasticity in typically developing children, and additionally demonstrate training-related white matter plasticity in preschool-aged children for the first time.

The white matter underlying the dorsal precentral gyrus, that is the area of white matter for which the word learning group showed a larger increase in FA than the two control groups, is a crossing zone of three major fiber bundles. The fibers of the corpus callosum, the cortico-spinal tract, and a fronto-parietal connection, the most dorsal branch of the SLF (Thiebaut de Schotten et al. 2011; Parlatini et al. 2017), course through this area. In previous studies, this left fronto-parietal region was found to show higher FA values in children with higher spatial working memory scores (Olesen et al. 2003; Nagy et al. 2004; Klingberg 2006). In adult training studies investigating white matter plasticity, a similar junction of crossing fibers including the fronto-parietal connection was reported in older adults as a result of memory training (de Lange et al. 2017) and multi-domain cognitive training (Cao et al. 2016). For the homologue fronto-parietal region in the right hemisphere, a change in FA was reported after reasoning training in young adults (Mackey et al. 2012).

In the current study, further evidence for the fronto-parietal change in FA being implicated in the word learning process is the observed correlation between FA change from scan 1 to scan 2 and the average training accuracy across all training sessions. We found that children who needed more training sessions to learn the pseudo-animal–label pairings showed a larger FA increase in the left postcentral white matter located directly posterior to the location of the group effect of FA change in the left precentral white matter. The vicinity of the location of these two effects in the pre- and postcentral white matter suggests the involvement of white matter structures beyond the local regions.

These left dorsal pre- and postcentral white matter regions as such are not directly part of the canonical language network. We propose that these white matter regions are part of the fronto-parietal connection of the left hemisphere supporting the increased use of domain-general processes crucial to word learning such as working memory and attention. Aside from being implicated in working memory, activation in these fronto-parietal regions has been linked to attention, and specifically top-down, goal-directed attention (Corbetta and Shulman 2002). In order for structural plasticity to occur, the particular task being trained should present a larger challenge for the system than what the system typically can handle (Lövdén et al. 2010). We interpret the lack of effects in the canonical language network in our study as evidence that the language aspect of the present training paradigm (word learning) did not challenge the children’s language network enough to induce change. Rather, the language network supporting lexical-semantic processes already appears to be well established, because word learning at the age of four years challenges domain-general systems instead of the language system as such.

Working memory, specifically phonological working memory, supports word learning by forming a precise memory trace. This allows reactivation upon repeated exposure, which in turn leads to a lasting lexical entry to be formed (Baddeley et al. 1998). In the present training sessions, which lasted 8–10 min, maintaining attention towards the task was important to achieve the goal of learning the presented words. In the paradigm used, however, we were unable to disentangle the two factors of working memory and attention and their respective impact on FA changes in the left fronto-parietal white matter. Due to the constraints in conducting longitudinal research in young children, it was not possible to collect additional behavioral measures after each training session, which could have provided further information concerning the impact of working memory and attention. Here, we can argue on the basis of the known brain-function relationship of the regions reported in the literature. Future studies are needed to discern the two aspects during word learning in children.

With respect to the language network, the present findings clearly demonstrate that word learning in preschool children is related to brain white matter structure in its ventral part. We found that white matter microstructure plays a predictive role for the behavioral outcome of word learning. Crucially, we report that white matter in the middle temporal cortex at the outset of the training predicts word learning. One white matter tract that courses through this region is the ILF which is part of the ventral language system linking temporal regions with inferior frontal regions via the uncinate fascicle. The ventral language stream has previously been related to semantic processing, both on the lexical as well as on the sentence level (Friederici and Gierhan 2013). Additionally, a meta-analysis distilling the regions of the brain involved in accessing knowledge from words reports the left middle temporal gyrus to be one of the key regions in this process (Binder et al. 2009). Our finding is in line with previous literature that draws a link between white matter microstructure and cognitive abilities in development in general (e.g. Deoni et al. 2016; Saygin et al. 2013; Vandermosten et al. 2015; Walton et al. 2018). Here, we demonstrate a direct relationship between FA of parts of the ventral language pathway and word learning ability in children, making our study the first to provide evidence for this phenomenon.

While one cannot associate one single biological mechanism to the change we found in FA, one possibility is myelination. Research on animal models can provide deeper insight into biological mechanisms that are not feasible in human research. Mice learning to do a complex motor task were unable to master this task when they were no longer able to produce new myelin (McKenzie et al. 2014), suggesting that myelination supports learning of complex tasks. MR sequences enabling non-invasive research in humans that can provide more detail on the biological underpinning of a change in white matter microstructure are available (e.g., Zhang et al. 2012), however, additional sequences also require more time spent in the MR scanner. This poses a problem when conducting research with very young children as in the present study, where the time spent in the scanner needs to be minimized to avoid motion artifacts.

In conclusion, here we report white matter microstructure changes in 4-year-old children following word learning. We found regional white matter change following novel word learning in the dorsally located structures which in adults are known to support domain-general top-down processes. In turn, the status of the ventrally located white matter structure in the left middle temporal gyrus before training was found to be predictive of early phases of word learning in the current experiment. This is the first investigation reporting word learning-related white matter changes in children, and furthermore, it is the first study to measure plasticity in typically developing children at the age of 4 years. Our findings suggest that in addition to the crucial role of semantic-related white matter structures in the temporal cortex, word learning is supported by a left fronto-parietal domain-general connection showing white matter structural changes as a function of training.

## Electronic supplementary material

Below is the link to the electronic supplementary material.

Supplementary file1 (PDF 99 kb)
