# Supplementary material for: Word learning reveals white matter plasticity in preschool children
Source: Brain Struct Funct. 2020 Feb 18;225(2):607–19. doi: 10.1007/s00429-020-02024-7 (PMC7046568; doi:10.1007/s00429-020-02024-7)
Supplement: Supplementary file 1 — Supplementary file1 (PDF 99 kb) [file 429_2020_2024_MOESM1_ESM.pdf]

Online Resource 1: Supplementary Figures

Word learning leads to white matter plasticity in preschool children

Brain Structure and Function

Clara E.M. Ekerdt, Clara Kühn, Alfred Anwander, Jens Brauer & Angela D. Friederici

Department of Neuropsychology, Max Planck Institute for Human Cognitive and Brain Sciences, Stephanstr. 1a, 04103 Leipzig, Germany

Address for correspondence: ekerdt@cbs.mpg.de

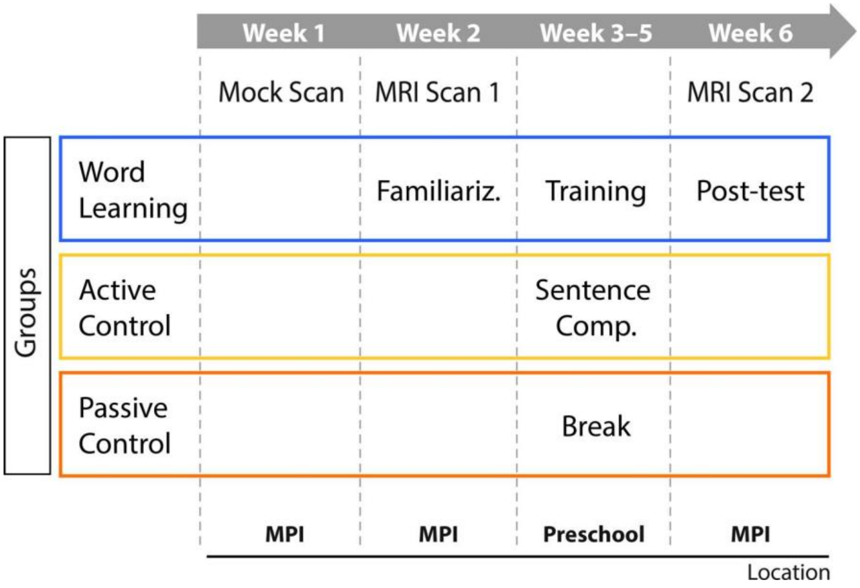

Supplementary Fig. 1 Timeline of study. IQ testing session is not shown in this timeline. IQ testing session took place six months after MRI Scan 1. MPI = research institute. At the time of scan 1 and scan 2, children in the active and passive control groups completed a sentence comprehension task after their MRI scan, as described in the Materials and Methods section.

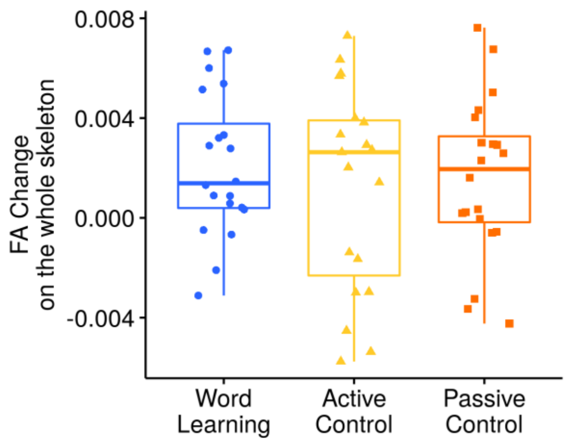

Supplementary Fig. 2 Mean FA change on the entire white matter skeleton from scan 1 to scan 2. The groups did not differ in global mean FA change from scan 1 to scan 2 on the white matter skeleton. Individual subjects' data points are represented by dots in the word learning group, triangles in the active, and squares in the passive control group.
